# Editorial: Integrative systems biology and big data for agricultural improvement and understanding

**DOI:** 10.3389/fsysb.2023.1347323

**Published:** 2023-12-20

**Authors:** Liliana Fadul-Pacheco, Antony T. Vincent, Eric R. Paquet

**Affiliations:** ^1^ Lactanet, Sainte-Anne-de-Bellevue, QC, Canada; ^2^ Département des Sciences Animales, Faculté des sciences de l’agriculture et de l’alimentation—Université Laval, Québec City, QC, Canada; ^3^ Institut de Biologie Intégrative et des Systèmes, Université Laval, Québec City, QC, Canada

**Keywords:** agriculture, maple syrup, dairy, swine, microbiome, forage

In the modern age, the agriculture industry faces the dual challenge of feeding an increasing global population and doing so sustainably. Meeting this challenge requires a more global understanding of biological systems. Integrative Systems Biology, a multidisciplinary approach that combines biology, genomics, and data analysis, has emerged as a powerful tool for improving agriculture and our comprehension of its biological components.

Agriculture has come a long way since the days of subsistence farming. Today, it is a complex web of interconnected factors, where crop yields, climate change, and environmental sustainability intersect. To navigate this complexity, Integrative Systems Biology allows us to study the intricate relationships within ecosystems, from the molecular level up to the farm scale. One pivotal facet of this paradigm shift is the exploration of microbiomes—complex communities of microorganisms that play a fundamental role in shaping the health, productivity, and sustainability of agricultural ecosystems. This Research Topic delves into the realm of systems biology with a keen focus on microbiomes, leveraging high-throughput sequencing technologies to unravel the mysteries hidden within diverse agricultural domains.

High-throughput sequencing, a revolutionary tool in genomics, has paved the way for a comprehensive exploration of microbial communities at unprecedented scales. This Research Topic embarks on an exploration journey across various agricultural landscapes, namely, forage systems, raw milk production, swine industry, and maple syrup production. Each of these fields represents a unique microcosm where the delicate balance of microbial interactions significantly influences the outcomes in terms of yield, quality, and overall system resilience.

This Research Topic aims to bridge the gap between traditional agricultural practices and cutting-edge molecular technologies, emphasizing the importance of systems biology and high-throughput sequencing in shaping the future of sustainable and resilient agricultural systems. Specifically, here is a summary of the content covered by the different research papers:

N’guyen et al. employs a systems biology approach, utilizing high-throughput amplicon sequencing and digital droplet PCR, to investigate the impact of dormancy release on microbial communities in maple sap. By identifying key microbial indicators and conducting predictive functional analysis, the research reveals a new unanticipated link between metabolic pathways and dormancy release. The findings highlight the significance of systems biology in understanding the complex interplay of microbial, vegetal, environmental, technological, and processing factors influencing the final composition of maple syrup.

Kennang Ouamba et al. examines the impact of commercial inoculants on dairy farm silage microbiota. High-throughput sequencing reveals differences between hay and silage, dominated by lactic acid bacteria. The inoculants, including *Lentilactobacillus buchneri*, inconsistently prevent undesirable bacteria in corn silage. Silage chemical profiles vary with sampling periods and inoculant use, and bacterial network analyses highlight variations in topological roles with inoculant application. The findings offer by the systems biology approach taken by the team provide insights for optimizing forage management and developing additives for improved hygienic quality and nutritional potential in preserved forage on dairy farms.

A second study by Ouamba et al., this time investigating the microbiota of bulk tank raw milk in relation to forage rations, including hay (H), grass/legume silage (GL), and combinations of grass/legume and corn silage with or without inoculation (GLC and GLICI). Three distinct community types were identified in forage rations, with differences in bacterial composition. Raw milk communities were influenced by forage types, with the GLC milk being phylogenetically different from GLICI. Approximately 18%–31% of amplicon sequence variants (ASVs) were shared between forage rations and corresponding raw milk, emphasizing the role of cow forage rations in raw milk contamination, and highlighting the relevance of this study for understanding on-farm raw milk microbiota.

Finally, Laforge et al. investigates the microbial ecology within the swine value chain, from animals to meat cuts, to optimize pork product microbiological quality. Distinctive microbiota profiles were identified in farms with different sanitary statuses. Despite similar bacterial counts at the meat plant, a reduction in microbiota diversity along the value chain was observed. Source tracker analysis indicated the potential for consistent microbiological quality from different farms, emphasizing the need for broader studies to assess the impact of herd sanitary status on final products.

In conclusion, this Research Topic clearly shows the pivotal role of integrative systems biology and big data in agriculture. The featured research papers highlight the transformative impact of these approaches across diverse agricultural domains, shedding light on the intricate relationships within ecosystems. By bridging traditional practices with cutting-edge molecular technologies, these studies pave the way for a more informed and sustainable future in agriculture, emphasizing the significance of understanding microbiomes and leveraging advanced genomics tools.

The guest editors would like to thank all the authors and reviewers for their work and devotion to the Research Topic, and hope that it can inspire further research in systems biology in agriculture.

